# Comparison of 2-octyl cyanoacrylate skin adhesive and interrupted polypropylene sutures for wound closure in total ankle arthroplasty

**DOI:** 10.1186/s13018-021-02791-x

**Published:** 2021-10-24

**Authors:** Gun-Woo Lee, Woo Kyoung Kwak, Keun-Bae Lee

**Affiliations:** Department of Orthopedic Surgery, Chonnam National University Medical School and Hospital, 42 Jebongro, Donggu, Gwangju, 61469 Republic of Korea

**Keywords:** Total ankle arthroplasty, Skin adhesive, Skin suture, Skin closure, Wound complication

## Abstract

**Background:**

Adhesive skin materials have increasingly been used in orthopedic surgery. We aimed to compare the efficacy and safety of skin adhesive (2-octyl cyanoacrylate and polymer mesh, Dermabond Prineo) and interrupted polypropylene sutures for wound closure in patients undergoing total ankle arthroplasty (TAA).

**Methods:**

We prospectively enrolled 107 consecutive patients (108 ankles) undergoing TAA and divided them into two groups: skin adhesive group (36 ankles) and suture group (72 ankles). The primary outcome assessment included wound complications and patient satisfaction for wound cosmesis. The secondary outcome assessment included duration of surgery, length of hospital stay, and the Ankle Osteoarthritis Scale (AOS) pain and disability score.

**Results:**

There was one case of allergic contact dermatitis, three cases of wound dehiscence, and one case of superficial surgical site infection in the skin adhesive group. Among them, one case each with allergic contact dermatitis and wound dehiscence finally progressed to deep surgical site infection. Three cases of wound dehiscence were also reported in the suture group; however, there was no case of surgical site infection. Patient satisfaction for wound cosmesis was significantly higher in the skin adhesive group than in the suture group (*p* = 0.001). There was no statistically significant difference between the groups in terms of secondary outcomes (*p* > 0.05).

**Conclusions:**

Although the use of Dermabond Prineo showed better patient satisfaction for wound cosmesis, it showed significantly high wound complication rates and no other clinical benefits compared to interrupted polypropylene suture in TAA. Our results suggest that awareness of the possibility of wound complications is necessary when Dermabond Prineo is used in TAA.

## Introduction

Total ankle arthroplasty (TAA) has been steadily developed while overcoming the drawbacks of the past and is widely performed as a primary treatment for end-stage ankle arthritis [1–9]. Wound closure is an important final step for good cosmesis and minimizing surgical site infection [10]. There is still no consensus about the best method of closing wounds in orthopedic surgery [11, 12]; however, skin suture methods consume a marked time during surgery with an added inconvenience of removal after healing [13]. In recent times, noninvasive skin closure methods including skin adhesive and zipper-like dressing are being widely used to minimize discomfort and scar formation [14–21].

Skin adhesive materials have become increasingly popular in elective orthopedic surgery [14, 16, 18–20, 22, 23]. The Dermabond Prineo topical skin adhesive (Ethicon, Somerville, NJ) consists of a 2-octyl cyanoacrylate liquid and a self-adhering polyester mesh with benzalkonium chloride. This product is known to reduce the risk of early infection after surgery by providing a water-tight seal to minimize wound drainage and an effective barrier to microorganisms [16].

Previous studies have reported the superiority of Dermabond Prineo skin adhesive in terms of cosmetic outcomes, lesser wound discharge, cost-effectiveness, and shorter length of hospital stay when used in hip and knee arthroplasty [15, 19, 20, 22, 23]. Despite these advantages and convenience in use, several recent studies have reported complications associated with its use; these are being increasingly reported as the use of the material has steadily increased. Most of these studies have highlighted concerns like allergic reactions following the use of Dermabond Prineo [10, 24–29]. This reaction is similar to postoperative infection and is difficult to distinguish; additionally, reaction symptoms are known to vary from mild to severe. Furthermore, in case of severe skin breakdown, it is highly likely to cause deep surgical site infection [10, 24].

Literature search showed no previous studies have evaluated the efficacy and safety of Dermabond Prineo as wound closure material in TAA. Thus, our study aimed to compare wound complication rate, duration of surgery, length of hospital stay, and clinical outcomes between Dermabond Prineo and interrupted polypropylene sutures used for wound closure in patients undergoing TAA.

## Materials and methods

### Patient selection

This study was approved by the institutional review board of our hospital. Between January 2016 and December 2018, 108 consecutive patients (111 ankles) underwent primary TAA using the mobile-bearing HINTEGRA prostheses (Newdeal, Lyon, France/Integra Lifesciences, Plainsboro, NJ USA). All operations were performed by one surgeon with experience of over 200 TAA.

The indication for primary TAA included end-stage ankle arthritis in patients with good general conditions, including well-controlled diabetes, good bone stock, and normal neurovascular status. The inclusion criteria for this study were symptomatic end-stage ankle osteoarthritis or rheumatoid arthritis with a minimum follow-up of 12 months after TAA. We excluded patients with hemophilic arthropathy and gouty arthritis of the ankle joint.

Finally, 107 patients (108 ankles) were enrolled and divided into two groups according to the method of wound closure. For the first half of the study period (from January 2016 to June 2017), 2-octyl cyanoacrylate and polymer mesh (Dermabond Prineo) were used in the skin adhesive group (36 patients, 36 ankles), and interrupted polypropylene suture was used during the second half of the study period from (July 2017 to December 2018) in the suture group (61 patients, 62 ankles).

### Surgical technique and removal of skin closure materials

All TAA were performed through the anterior longitudinal approach between the extensor hallucis longus and tibialis anterior tendon under general or spinal anesthesia. Distal tibial and talar dome resections were performed perpendicular to the mechanical axis of the tibia. After the medial, lateral, and posterior talar cuts, appropriately sized prostheses were implanted. If necessary, concomitant bony or ligamentous procedures were performed to achieve neutral hindfoot alignment and ligamentous balance.

In all patients, the anterior joint capsule and extensor retinaculum were repaired using 1–0 braided absorbable sutures (Vicryl, Ethicon, Somerville, NJ). The subcutaneous layer was repaired using 2–0 monofilament absorbable suture (Monosyn, B. Braun, Rubi, Spain). For skin closure, Dermabond Prineo was applied, followed by air drying for 1 min in the skin adhesive group, while 3–0 nonabsorbable polypropylene (Prolene, Ethicon, Somerville, NJ) horizontal mattress sutures were performed in the suture group. Subsequently, a compressive dressing and posterior short leg splint were applied in neutral ankle position. All skin closure materials were removed between 10 and 12 days after surgery. Particularly, for the removal of Dermabond Prineo, petroleum jelly (Vaseline, Unilever) was applied to loosen it, and then it was carefully peeled off from the skin. After removing suture materials, the patients were allowed to begin a range-of-motion exercise. Four weeks after surgery, the patient was instructed to begin progressive weight-bearing by wearing an ankle–foot orthosis. Full weight-bearing ambulation without orthosis began 6 to 8 weeks after surgery.

### Clinical evaluation

Clinical assessment included the wound complications, patient satisfaction for wound cosmesis, duration of surgery, hospital stay, and Ankle Osteoarthritis Scale (AOS) pain and disability score. The incidence of wound complications and patient satisfaction for wound cosmesis were the primary assessment parameters. Wound complications were evaluated during hospitalization or at the outpatient clinic 4 weeks after surgery, and the satisfaction survey was conducted at least 12 months after surgery. The wound complications were subdivided into allergic contact dermatitis (ACD), wound dehiscence, and surgical site infection (SSI). The appearance of an eczematous eruption or blister at the suture area or the area where the skin adhesive mesh was attached was classified as ACD  [29]. The wound dehiscence is defined as a partial or total separation of previously approximated wound edges due to a failure of proper wound healing [30]. The SSI  was classified into superficial or deep infection based on the extent of infection [31]. Patient satisfaction was surveyed through a questionnaire administered to the patients and categorized into ‘very satisfied,’ ‘satisfied,’ ‘as expected,’ and ‘dissatisfied.’

The secondary assessment included duration of surgery, length of hospital stay, and functional outcomes. Functional outcome was evaluated before surgery, at 6 and 12 months after surgery, and every year thereafter by AOS pain and disability score, which was reliable and validated for ankle joint-specific outcome [32].

### Statistical analyses

Data were analyzed using SPSS (version 23.0, IBM Corp., Armonk, NY USA). Descriptive statistics were calculated using the standard formulae. Kolmogorov–Smirnov test was used to verify the normal distribution of data variables. An independent *t* test was used to analyze the differences between the groups for normally distributed continuous variables; else, Mann–Whitney *U* test was used for comparing variables that were not normally distributed. For the categorical variables, Chi-square or Fisher’s exact tests were used to analyze the differences. All statistical analyses were reviewed by a statistician, and a *p*-value < 0.05 was considered significant.

## Results

The information of patients in each group is shown in Table 1. There were no significant differences in age, sex, presence of diabetes, number of current smokers and antithrombotic drug users, body mass index (BMI), preoperative diagnosis, follow-up period, and other medical conditions (*p* > 0.05) between the groups.

**Table 1:** Patient demographics

	Skin adhesive group (*N* = 36 ankles)	Suture group (*N* = 72 ankles)	*p*-value*
Age (years)†	67.7 ± 7.1 (53 to 80)	67.1 ± 8.3 (49 to 86)	0.731
Sex‡			0.785
Male	18 (50.0%)	33 (45.8%)	
Female	18 (50.0%)	39 (54.2%)	
Diabetes‡	6 (16.7%)	13 (18.1%)	0.726
Current smoker‡	2 (5.6%)	8 (11.1%)	0.491
Antithrombotic drug use‡	4 (11.1%)	8 (11.1%)	0.999
BMI (kg/m^2^)†	26.5 ± 3.7 (18.1 to 34.3)	25.4 ± 2.9 (18.6 to 32.0)	0.163
Hemoglobin (g/dL)	13.9 ± 1.4 (11.1 to 17.2)	13.7 ± 1.4 (9.3 to 17.7)	0.484
Albumin (g/dL)	4.4 ± 0.3 (3.8 to 5.1)	4.4 ± 0.3 (3.7 to 5.2)	0.876
Platelets (10^3^/μL)	236.2 ± 61.3 (121.0 to 372.0)	239.7 ± 60.9 (141.0 to 445.0)	0.794
Diagnosis‡			0.286
Primary osteoarthritis	21 (58.3%)	39 (54.2%)	
Posttraumatic osteoarthritis			
Postfracture	–	7 (9.7%)	
Recurrent ankle sprain	14 (38.9%)	24 (33.3%)	
Rheumatoid arthritis	1 (2.8%)	2 (2.8%)	
Follow-up (month)†	38.7 ± 7.1 (18 to 48)	35.3 ± 10.7 (20 to 61)	0.069

Abbreviations: *BMI*, body mass index

^*^The independent *t* test was used to analyze differences in age, BMI, hemoglobin, albumin, platelets, and follow-up duration. The Chi-square or Fisher’s exact test was used to analyze differences in sex, diabetes, current smoker, antithrombotic drug use, and preoperative diagnosis between the groups. A *p*-value of < 0.05 was considered significant

^†^The values are given as the mean ± standard deviation, with the range in parentheses

^‡^The values are given as the number of ankles, with the percentage in parentheses

The incidence of wound complications is shown in Table 2. The overall number of ankles with wound complications was 5 (13.9%) of 36 cases in the skin adhesive group and 2 (2.8%) of 72 cases in the suture group (*p* = 0.04). There was one case of allergic contact dermatitis, three cases of wound dehiscence, and one case of superficial surgical site infection in the skin adhesive group. Two cases of wound dehiscence were also reported in the suture group; however, there was no case of surgical site infection.

**Table 2:** Wound complications following total ankle arthroplasty according to the wound closure method

	Skin Adhesive Group (*N* = 36 ankles)	Suture group (*N* = 72 ankles)	*p*-value†
None	31 (86.1%)	70 (97.2%)	0.040
Allergic contact dermatitis	1 (2.8%)	–	0.333
Wound dehiscence	3 (8.3%)	2 (2.8%)	0.331
Surgical site infection			0.035
Superficial	1 (2.8%)	–	
Deep	2^‡^ (5.6%)	–	

The values are given as the number of ankles, with the percentage in parentheses

^†^Fisher’s exact test was used to analyze the intergroup differences. A *p*-value of < 0.05 was considered significant

^‡^The overall number of ankles with wound complications was 5 (13.9%) of 36 cases in the skin adhesive group. Among them, one case each of allergic contact dermatitis and wound dehiscence progressed to deep infection

**Fig. 1:**
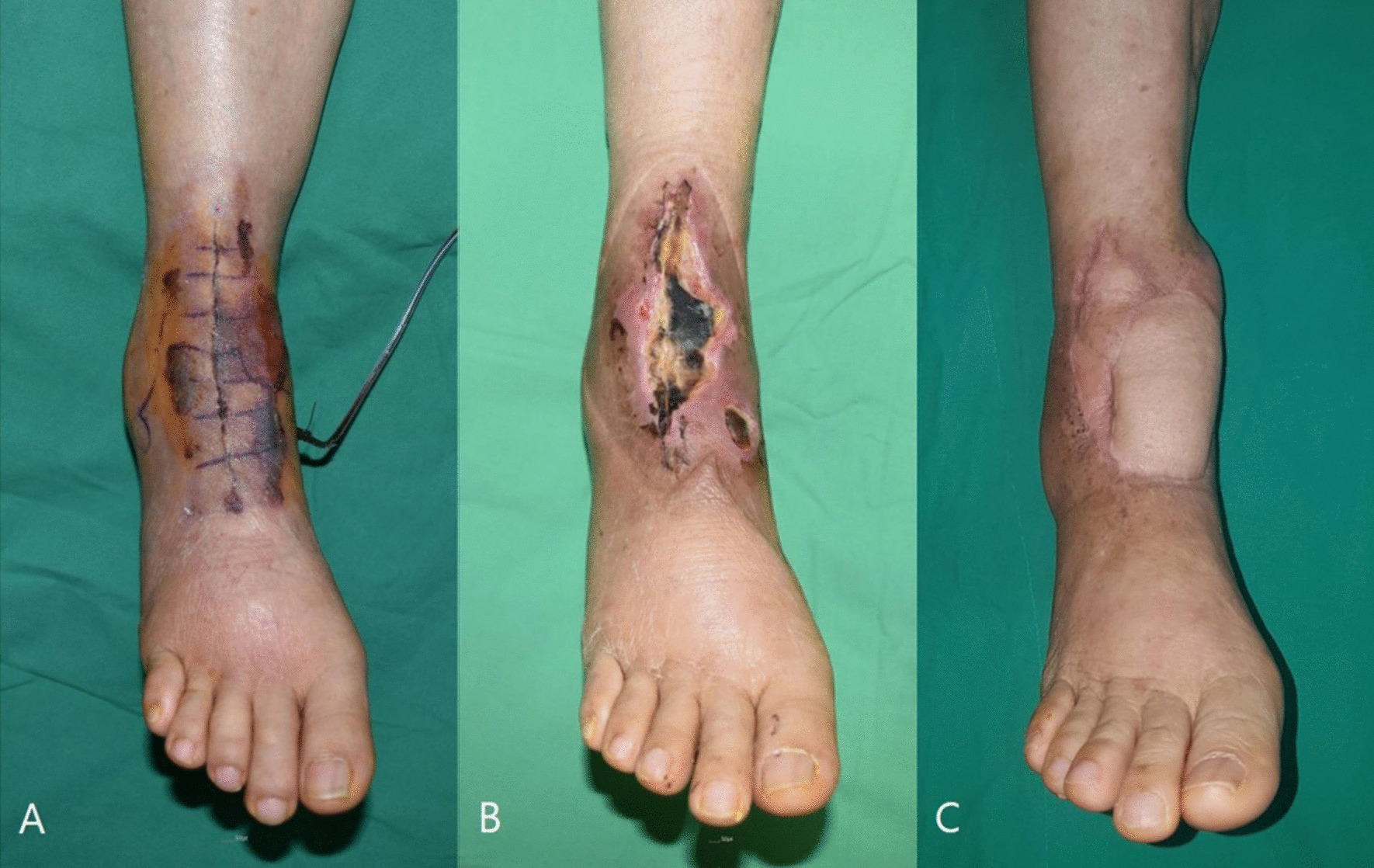
**a** A 64-year-old female developed allergic contact dermatitis (ACD) after applying Dermabond Prineo for wound closure. **b** Despite wound care using negative pressure wound therapy (NPWT) and prolonged intravenous antibiotic treatment, she developed deep surgical site infection (SSI) with wound necrosis. **c** The patient recovered after repeated debridement and polyethylene liner exchange without implant removal; in addition, a local flap surgery was performed for the accompanying soft tissue necrosis

**Fig. 2:**
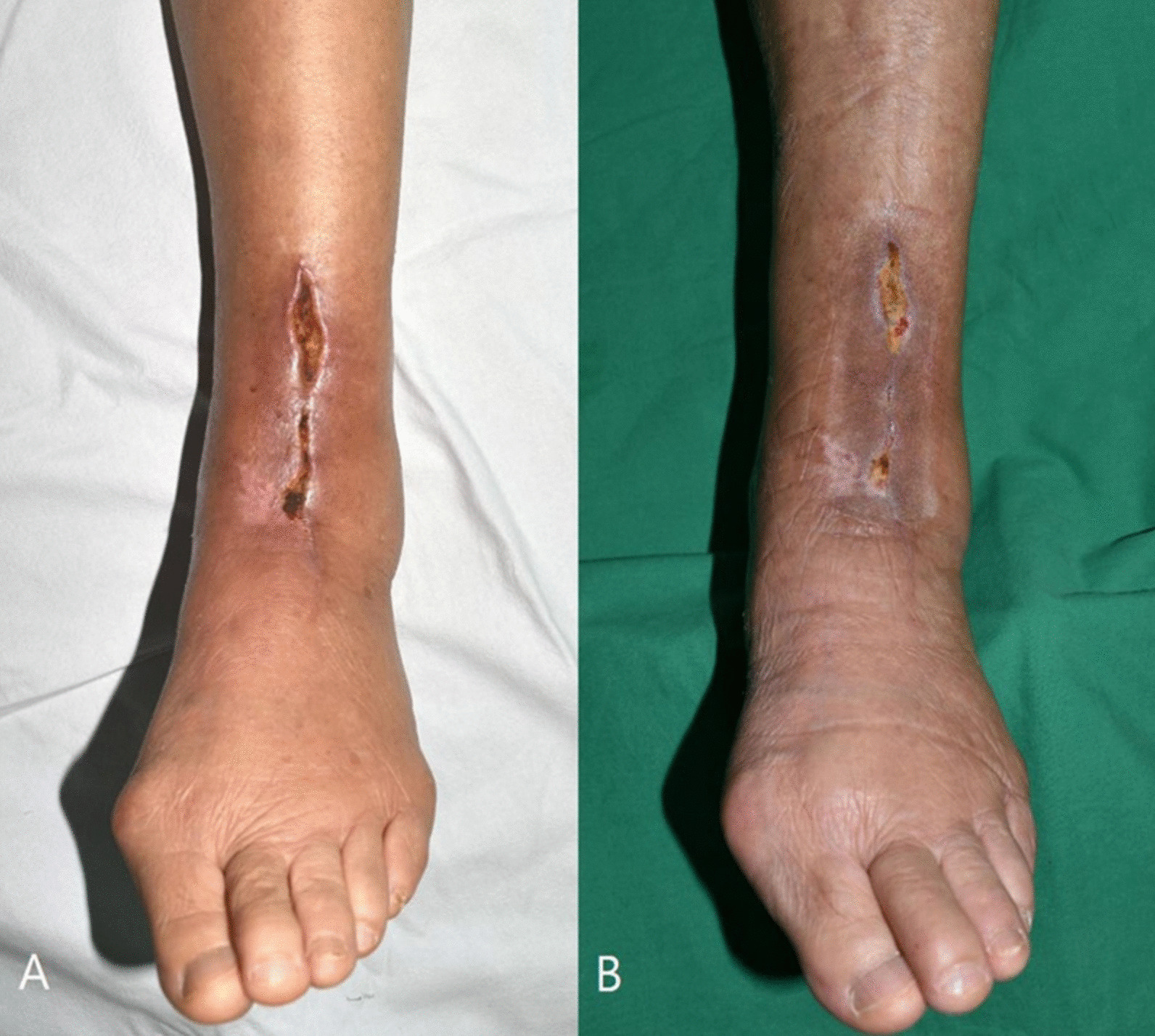
**a** A 75-year-old female patient developed full incisional wound dehiscence after wound closure with Dermabond Prineo. **b** The wound recovered by applying negative pressure wound therapy (NPWT) without the need for further surgery

One case of ACD and 3 cases of SSI were seen in the skin adhesive group, whereas these complications did not occur in the suture group (Fig. 1). Among them, one case with superficial SSI improved with antibiotic treatment and wound care without further surgery. However, one case, each of ACD and wound dehiscence progressed to deep SSI. The patient who developed ACD followed by deep SSI recovered after repeated debridement and polyethylene liner exchange without implant removal; however, a local flap surgery was performed for the accompanying soft tissue necrosis. The patient with deep SSI due to wound dehiscence was successfully treated by revision TAA in a two-stage procedure using an antibiotic-impregnated cement spacer. The 4 cases of wound dehiscence (2 in the skin adhesive group, 2 in the suture group) healed with daily dressing and prolonged antibiotic use. There was a case with full incisional dehiscence, which did not occur after skin suture in the skin adhesive group (Fig. 2).

Regarding patient satisfaction for wound cosmesis, 34 (94.4%) of 36 patients in the skin adhesive group reported that they were ‘very satisfied’ or ‘satisfied,’ and there was a significant difference compared to the responses in the suture group (Table 3).

**Table 3:** Patient satisfaction for wound cosmesis following total ankle arthroplasty according to the wound closure method

	Skin adhesive group (*N* = 36 ankles)	Suture group (*N* = 72 ankles)	*p*-value†
Level of satisfaction			0.001
Very satisfied	30 (83.3%)	32 (44.4%)	
Satisfied	4 (11.1%)	32 (44.4%)	
As expected	–	8 (11.2%)	
Dissatisfied	2 (5.6%)	–	

The values are given as the number of ankles, with the percentage in parentheses

^†^Fisher’s exact test was used to analyze the intergroup differences. A *p*-value of < 0.05 was considered significant

The comparison of duration of surgery, length of hospital stay, and AOS pain disability score is shown in Table 4. There was no statistically significant difference in the duration of surgery and length of hospital stay between the two groups (*p* > 0.05). In terms of the functional outcomes, there were no significant intergroup differences in the AOS pain, and disability scores at the final follow-up (*p* > 0.05).

**Table 4:** Other clinical outcomes following total ankle arthroplasty according to the wound closure method

	Skin adhesive group (*N* = 36 ankles)	Suture group (*N* = 72 ankles)	*p*-value*
Duration of surgery (min)†	113.4 ± 23.8 (70 to 160)	114.1 ± 22.1 (75 to 160)	0.918
Hospital stay (day)‡	11.0 (8.0 to 14.0)	13.0 (9.0 to 15.0)	0.239
AOS pain score†			
Preoperative	58.0 ± 14.3 (33.3 to 85.7)	54.7 ± 17.1 (21.4 to 82.9)	0.437
Final	29.2 ± 20.4 (0.0 to 80.0)	24.4 ± 18.0 (0.0 to 71.4)	0.288
AOS disability score†			
Preoperative	68.8 ± 16.1 (21.1 to 95.6)	71.3 ± 16.9 (34.4 to 93.3)	0.558
Final	37.6 ± 18.2 (6.7 to 80.0)	31.0 ± 19.4 (0.0 to 82.2)	0.148

Abbreviations: *AOS*, Ankle Osteoarthritis Scale

^*^The independent *t* test was used to analyze differences in duration of surgery, AOS pain and disability scores. The Mann–Whitney U test was used to analyze differences in hospital stay. A *p*-value of < 0.05 was considered significant

^†^The values are given as the mean ± standard deviation, with the range in parentheses

^‡^The values are given as the median, with the interquartile range in parentheses

## Discussion

To our knowledge, this is the first study to evaluate the efficacy and safety of Dermabond Prineo for skin closure after TAA. The most important finding of this study is that the use of Dermabond Prineo showed a significantly high rate of overall wound complications and did not show any other benefits in terms of duration of surgery, length of hospital stay, and clinical outcomes.

In recent times, skin adhesive materials, including Dermabond Prineo, are widely used in elective orthopedic surgery due to various advantages [14, 15, 18–22, 33]. These benefits are still debatable and recent studies have repeatedly reported concerns about wound complications following the use of a skin adhesive material composed of 2-octyl cyanoacrylate [27, 34–37]. Almustafa et al. investigated the risk factors for SSI by analyzing 2,100 cases of primary total knee arthroplasty. The use of 2-octyl cyanoacrylate skin glue for wound closure was identified as a risk factor for SSI following total knee arthroplasty [38]. With respect to ankle surgery, Park et al. demonstrated that the use of 2-octyl cyanoacrylate topical skin adhesive (Surgiseal, Adhezion Biomedical LLC, Wyomissing, PA, USA) for wound closure was effective and safe with high patient satisfaction after ankle fracture surgery [18]. However, they also reported that the attention was needed because the statistical power of the complication was insufficient.

In this study, there was a case of full incisional dehiscence, which did not occur in the suture group. We assumed that tension around the entire wound might have led to the occurrence of full wound dehiscence. In the case of a skin suture, the tension is applied focally around the skin through which the thread passes; however, the tension is applied to the wide area of the entire wound when skin adhesive is used. Other previous studies have also described that increasing tissue tension can lead to superficial skin damage by shearing the skin when the skin adhesive material was used [38]. Furthermore, there were two cases of deep SSI in the skin adhesive group, of which one each progressed from ACD and wound dehiscence. We assumed that the relatively thin soft tissue envelope of the ankle joint compared to that of the hip or knee joint might increase vulnerability to the development of SSI when ACD or wound dehiscence occurs.

Based on our experience of wound complications in the skin adhesive group, we have discontinued the use of Dermabond Prineo for wound closure in TAA. Since there are not many studies on the use of Dermabond Prineo in ankle surgery, it is too early to conclude that its use increases the rates of wound complication and SSI. However, it has been frequently reported that the constituents of Dermabond Prineo induce allergic reactions. These can lead to ACD or wound dehiscence and can cause critical complications such as deep SSI [10, 25, 27–29, 34–37, 39–42]. ACD is more likely to develop when the skin is in prolonged contact with the allergen or when the amount of allergen is high [36, 37, 39].

There is still no established method for the prophylaxis of ACD after the use of Dermabond Prineo. According to previous reports, patch testing for Dermabond Prineo glue or polyester mesh has been known to be useful to identify the patient who may have allergenic reactions postoperatively [29]. Bulky occlusive dressings after TAA can increase the absorption of allergens, and water or a humid environment can cause depolymerization to create a strong sensitizer, a monomer. Thus, dry permeable dressing is recommended to minimize an allergic reaction [34, 43, 44]. In addition, the application of incisional negative pressure wound therapy (NPWT) has been known to reduce the incidence of wound complications after TAA. However, the efficacy and safety of NPWT after the use of Dermabond Prineo have not yet been reported [45–47].

This study had several weaknesses. First, the sample size of both groups was small. This limited our ability to evaluate the safety of Dermabond Prineo. Second, we did not measure the time skin taken for closure separately and evaluated the cosmetic outcome by the objective wound score. Finally, we did not perform preoperative testing for other allergens that could cause skin complications. We use chlorhexidine for preoperative skin preparation, which is also known to be an allergen.

## Conclusion

In conclusion, although the use of Dermabond Prineo showed better patient satisfaction for wound cosmesis, there was a significantly high rate of overall wound complications, and there were no other benefits in terms of duration of surgery, length of hospital stay, and clinical outcomes compared to those with the use of interrupted polypropylene suture in TAA. Our results suggest that careful vigilance is necessary when using Dermabond Prineo in TAA. Further studies with larger samples are necessary to determine the effect of Dermabond Prineo in wound closure.

## Data Availability

The datasets analyzed during the current study are not publicly available due to patient confidentiality.

## Abbreviations

TAA: Total ankle arthroplasty
AOS: Ankle Osteoarthritis Scale
ACD: Allergic contact dermatitis
SSI: Surgical site infection
BMI: Body mass index
